# 

**Published:** 1910-05-21

**Authors:** 


### Gifts and Bequests.

Queen Charlotte's Lying-in Hospital has received a grant of £10 10s. from the Skinners' Company.

The treasurers of the Middlesex Hospital have received a further grant of £210 from the Mercers' Company.

The Infants' Hospital, Vincent Square, Westminster, has received a donation of ten guineas from the Skinners' Company.

The Council of the Friedenheim Hospital, Swiss Cottage, N.W., acknowledges a donation of £10 10s. from the Skinners' Company.

The Chelsea Hospital for Women has received from the Mercers' Company a grant of £26 5s., and from the Salters' Company £5 5s. (annual subscription).

The Treasurer of St. Bartholomew's Hospital acknowledges the receipt of the following further contribution towards the special fund: Messrs. Speyer Brothers, £500.

Sir Walter Palmer, a director of Messrs. Huntley and Palmers, Ltd., who died on April 16, aged 52, left £200 to the Royal Free Hospital, Gray's Inn Road, W.C., and £100 to the Royal Berkshire Hospital, Reading.

Mr. Charles Smith, of Carlton Hill, St. John's Wood, N.W., who died on April 19, left £1,000 each to St. Mary's, St. George's, St. Bartholomew's, St. Thomas's, Guy's, and University College Hospitals, Charing Cross, Middlesex, and King's College Hospitals, the Royal Free Hospital, Cancer Hospital, Fulham Road, S.W., the Brompton Hospital for Consumption, and the Samaritan Free Hospital for Women; £750 to the British Medical Benevolent Fund, 15 Wimpole Street, W.; and £500 each to the Hospital for Paralysis, Maida Vale; Hospital for Children, Paddington Green, W.; Royal Ear and Throat Hospital, Dean Street, Soho; Home for the Dying, Avenue Road, Swiss Cottage, N.W.; Marylebone Almshouses, St. John's Wood Terrace, N.W.; Hospital for Sick Children, Great Ormond Street; British Hospital for Diseases of the Skin, Queen Charlotte's Lying-in Hospital, and Cripples' Home and Industrial School, Marylebone Road.

Mr. Frederick Tendron, of the Manor House, Bishopsdown, Tunbridge Wells, chairman of the Assam Company and of the St. John del Rey Mining Company, Ltd., who died on April 14, aged 76, left £1,000 each to the National Society for the Prevention of Cruelty to Children, the Royal Society for the Prevention of Cruelty to Animals, Dr. Barnardo's Homes, the National Refuges for Homeless and Destitute Children, 164 Shaftesbury Avenue, W.C., the London Hospital for General Purposes, and the Poplar Hospital for Accidents; and £500 to the Destitute Children's Dinners Society. The residue of his property, which apparently amounts to about £35,000, he left equally between the London Hospital, the Tunbridge Wells General Hospital, and the Society for the Relief of the Distressed, 78 Jermyn Street, S.W.

### Jottings.

The matinée announced to take place at the Palace Theatre, Leicester, on Thursday, May 19, will take place on the 26th inst. By the kindness of Mr. Oswald Stoll, principal, artistes from Sheffield, Nottingham, Leeds, Birmingham, and surrounding halls will support the programme. The whole of the proceeds of the matinée (without any deduction whatever) will be devoted to the institution.

The bazaar in aid of the Hospital for Women, Soho Square, will take place, as originally arranged, on May 24 and 25, 1910, in the new hospital buildings. The concert in this connection on the latter date will, it is hoped, be a great success. The artistes who have promised their help include the Earl of Shaftesbury, K.C.V.O., the Hon. Mrs. Stuart Anderson, Madame Louise Dale, Mrs. George Swinton, Mr. C. Hayden Coffin, Mr. Maurice Farkoa, and Miss Lena Ashwell. There are very few tickets left to be disposed of. The bazaar is being promoted with the object of raising £4,500 to complete the Rebuilding Fund, and to put the hospital in a position to claim a handsome conditional gift of £3,000 from King Edward's Hospital Fund. The stallholders include the Countess of Shaftesbury, the Countess of Strafford, the Countess of Ranfurly, Evelyn Countess Bathurst, the Viscountess Clifden, Baroness Anthony de Worms, the Lady Mary Scott, the Lady Evelyn Ewart, the Lady Mersey, Lady Kathleen Brownlow, Lady Maxwell Lyte, and Lady Stern.

THE

### GRADUATES' MEDICAL SCHOOLS.

DIARY FOR THE WEEK, MAY 23 to MAY 28.

**ROYAL SOCIETY OF MEDICINE,** 15 Cavendish Square, W. (Temporary address during building.)

At 5 p.m.—**May 23,** Special Meeting of Fellows of the Society.

At Morley Hall, George Street, Hanover Square, W.

*Discussion* on "Vaccine Therapy; its administration, value, and limitations," opened by Sir Almroth Wright, F.R.S. At this, the first meeting, the following gentlemen will continue the discussion: Dr. C. Slater, Dr. L. Colebrook, Dr. St. Clair Thomson, and Dr. W. d'Este Emery.

8 p.m.—**May 23, Odontological Section:**

*Paper*: Mr. Douglas E. Caush: "The spindles of Von Ebner" (illustrated by lantern slides).

*Casual communications*: Mr. Arthur S. Underwood: "Some Egyptian skulls." Mr. G. Thomson: "A case showing the result of extraction of the six-year-old molars."

5.30 p.m.—**May 24, Medical Section:**

Annual General Meeting: Election of officers and Council for session 1910-11. Paper.

5 p.m.—**May 25,** Special Meeting of Fellows of the Society.

At Morley Hall, George Street, Hanover Square, W.

*Discussion* on "Vaccine Therapy; its administration, value, and limitations," reopened by Dr. Hale White, and continued by Dr. J. W. H. Eyre, Sir W. Watson Cheyne, Bart., C.B., F.R.S., Dr. John Matthews, Dr. Harold A. Des Vœux, Dr. H. W. Bayly, Dr. A. Butler Harris.

7.45 p.m.—**May 26, Obstetrical and Gynæcological Section:**

Annual General Meeting: Election of officers and Council for session 1910-11.

*Papers*: Dr. F. J. McCann: "Cæsarean section in the treatment of eclampsia gravidarum." Dr. A. J. Wallace: "Contribution to the life-history of fibro-myomata of the uterus."

4.30 p.m.—**May 27, Section for the Study of Disease in Children:**

At 11 Chandos Street, W.

*Cases*: Dr. E. Bellingham Smith: Amaurotic family idiocy, and other cases.

5 p.m.—**May 27,** General Meeting of Fellows: Election of Candidates for Fellowship.

At 15 Cavendish Square, W.

6 p.m.—**May 27, Section of Anæsthetics:**

Annual General Meeting: Election of officers and Council for session 1910-11.

8.30 p.m.—**May 27, Epidemiological Section.**

**May 28, Section of Balneology and Climatology:**

**ST. MARK'S HOSPITAL FOR DISEASES OF THE RECTUM,** City Road, E.C.

At 2.30 p.m.—**May 27,** Mr. Aslett Baldwin, **Piles.**

**THE POST-GRADUATE COLLEGE,** West London Hospital, Hammersmith, W.

At 10 a.m.—**May 23** and **26,** Demonstrations by Surgical Registrar.

10 a.m.—**May 27,** Demonstration by Medical Registrar.

11.30 a.m.—**May 24,** Demonstrations of Minor Operations.

12.15 a.m.—**May 25,** Dr. Grainger Stewart, **Practical Medicine.**

5 p.m.—**May 23,** Mr. Armour, **Operations for Appendicitis.**

5 p.m.—**May 24,** Dr. Low, **Insects as Carriers of Disease in Tropical Medicine.**

5 p.m.—**May 25,** Dr. Bernstein, **What can be Learnt from a Blood Examination.**

5 p.m.—**May 26,** Mr. Baldwin, **Practical Surgery.** Lecture I.

5 p.m.—**May 27,** Dr. Pritchard, **Clinical Pathology.**

**LONDON SCHOOL OF CLINICAL MEDICINE,** Seamen's Hospital, Greenwich, S.E.

At 3.15 p.m.—**May 24,** Mr. Cosling, **Cancer of the Intestines.**

2.15 p.m.—**May 27,** Dr. R. Bradford, **Chronic Peritonitis.**

**THE BROMPTON HOSPITAL FOR CONSUMPTION AND DISEASES OF THE CHEST,** Fulham Road, S.W.

At 4 p.m.—**May 25,** Dr. Acland, **Inflammatory Lesions in the neighbourhood of the Diaphragm.**

**NORTH-EAST LONDON POST-GRADUATE COLLEGE,** Prince of Wales's General Hospital, Tottenham, N.

**May 24,** Dr. G. P. Chappel, **Selected Medical Cases.**

**May 26,** Mr. H. W. Carson, **Surgical Diseases of the Pancreas.**

**MEDICAL GRADUATES' COLLEGE AND POLYCLINIC.** 22 Chenies Street, W.C.

At 4 p.m.—**May 23,** Dr. T. Colcott Fox, **Clinique (skin).**

5.15 p.m.—**May 23,** Mr. H. J. Paterson, **The Simulation of Gastric Disease by Chronic Appendicitis.**

4 p.m.—**May 24,** Dr. Hector Mackenzie, **Clinique (medical).**

5.15 p.m.—**May 24,** Mr. A. H. Tubby, **Treatment of Lateral Curvature of the Spine in Children.**

4 p.m.—**May 25,** Mr. E. Laming Evans, **Clinique (surgical).**

5.15 p.m.—**May 25,** Dr. James Collier, **Myelitis.**

4 p.m.—**May 26,** Dr. C. O. Hawthorne, **Clinique (medical).**

5.15 p.m.—**May 26,** Dr. George Pernet, **Lupus Erythematosus.**

4 p.m.—**May 27,** Dr. Dundas Grant, **Clinique (Ear, Nose and Throat).**

**THE HOSPITAL FOR SICK CHILDREN,** Great Ormond Street, W.C.

At 4 p.m.—**May 26,** Mr. Kellock, **Demonstration of Selected Surgical Cases.**

